# Simultaneous ^13^N-ammonia and gadolinium perfusion using integrated PET-MRI: diagnostic accuracy in coronary microvascular dysfunction

**DOI:** 10.3389/fmed.2025.1649175

**Published:** 2025-09-01

**Authors:** Runze Wen, Qiang Xie, Bo Pan, Ming Ni, Xingxing Zhu, Xueer Meng, Zhenheng Wei, Xinai Wu, Dan Li, Xuemei Wang

**Affiliations:** ^1^Department of Nuclear Medicine, First Affiliated Hospital of USTC, Division of Life Sciences and Medicine, University of Science and Technology of China, Hefei, Anhui, China; ^2^Department of Nuclear Medicine, Affiliated Hospital of Inner Mongolia Medical University, Hohhot, Inner Mongolia, China; ^3^Department of Cardiovascular Medicine, First Affiliated Hospital of USTC, Division of Life Sciences and Medicine, University of Science and Technology of China, Hefei, Anhui, China

**Keywords:** myocardial perfusion, myocardial blood flow, magnetic resonance imaging, positron emission tomography, coronary flow reserve

## Abstract

**Objective:**

Absolute quantification of myocardial perfusion and coronary flow reserve (CFR) with positron emission tomography (PET) has demonstrated diagnostic and prognostic value in patients with coronary microvascular dysfunction (CMD). However, no studies have compared magnetic resonance imaging (MRI) and PET perfusion imaging in patients with CMD using integrated PET-MRI imaging. The aim of this study was to assess the quantitative accuracy of cardiac perfusion measurements using MRI with simultaneous ^13^N-ammonia PET as reference with a fully integrated PET-MRI scanner.

**Methods:**

Thirty patients with suspected CMD underwent simultaneous MRI and ^13^N-ammonia PET scans at rest and regadenoson-stress on an integrated PET-MRI scanner. Correlation and agreement between MRI-and PET-derived myocardial blood flow (MBF) and CFR values were evaluated using correlation and Bland–Altman analysis.

**Results:**

MRI measurements of global rest and stress MBF showed moderate correlation to those obtained using ^13^N-ammonia PET (*r* = 0.50; *p* = 0.005 for rest MBF and *r* = 0.49; *p* < 0.006 for stress MBF). Bland–Altman analysis revealed a mean bias of −0.83 ± 0.47 mL/g/min for rest MBF and −1.84 ± 0.57 mL/g/min for stress MBF. The correlations between regional MBF_MRI_ and MBF_PET_ obtained during rest and stress were only poor to moderate (*r* = 0.26 and *r* = 0.43). The limits of agreement were wide for both global and regional MBF, with larger variability for high MBF-values. However, there was good agreement between MRI and PET with regard to global and regional CFR with moderate to strong correlation (*r* = 0.64, *p* < 0.001; *r* = 0.48, *p* < 0.001). MRI-derived CFR demonstrated an area under the curve (AUC) of 0.85 (95% CI: 0.67 to 0.95) and had an optimal cutoff value of 1.57 for detecting CMD, defined as ^13^N-ammonia PET-derived CFR ≤ 2.0.

**Conclusion:**

CFR measurements were concordant between MRI and ^13^N-ammonia PET. For detecting significant CMD, CFR_MRI_ and CFR_PET_ demonstrated comparable and high accuracy. Nevertheless, MRI measurements of rest and stress MBF showed only poor to modest agreement to those obtained with ^13^N-ammonia PET. Therefore, although quantitative MRI has clinical utility, further refinements are still required.

## 1 Introduction

Microvascular disease (MVD), or coronary microvascular dysfunction (CMD) is receiving increasingly attention as 20 to 80% of patients with stable angina have normal or nonobstructive coronary artery disease (NOCAD) on coronary angiography (CAG) and coronary computed tomography angiography (CCTA) (1). Current evidence suggests that CMD is multifactorial, resulting from both impaired vasodilation of the myocardial microcirculation and an increased response to vasoconstrictive stimuli (2). Consequently, the coronary microcirculation fails to vasodilate for maintaining normal myocardial perfusion (3).

With the growing recognition that CMD represents a potential therapeutic target, accurate diagnosis of this condition has taken on greater significance. However, the diagnosis of CMD has traditionally been ascertained by means of invasive physiological assessment during angiography but this method requires an invasive pressure measurement and thus carries increased risk to patients compared to noninvasive imaging (4). In recent years, there has been increasing interest in using non-invasive modalities, including positron emission tomography (PET), to assess coronary flow reserve (CFR) as the primary criterion for CMD (5). PET provides a non-invasive method to diagnose CMD through absolute quantification of hyperemic myocardial blood flow (MBF, mL/min/g) and CFR. Reductions in these parameters provide important diagnostic and prognostic information (6). However, the use of PET involves ionizing radiation exposure, which poses challenges for routine follow-up assessments.

In clinical routine and in large clinical studies, first-pass cardiac magnetic resonance imaging (MRI) has become a clinical standard approach to assess myocardial perfusion (7–9). Derived from time-intensity or contrast concentration curves obtained from the left ventricle (LV) tissue and blood pool, the myocardial perfusion reserve index (MPRI) serves as a reliable semiquantitative imaging marker reflecting the vasodilatory capacity of the microvasculature (10). Despite its promise, this technique still lacks clinical validation against an independent reference standard.

Previous studies showed good agreement between PET and cardiac magnetic resonance imaging (CMR) for myocardial perfusion reserve (stress-to-rest MBF ratio) (11, 12). Nevertheless, numerous studies comparing MBF and/or CFR between CMR and PET have primarily focused on separate assessments of these modalities performed on common groups of subjects but separated in time and thus being subject to intrasubject variation (11, 13, 14).

Therefore, the aim of this study was to evaluate the agreement between CMR and ^13^N-ammonia PET perfusion measurements using integrated PET-MRI imaging in a relatively large cohort of patients with CMD.

## 2 Methods

### 2.1 Study population

Data of this single-center study were derived from ongoing PET-MRI research projects. Thirty patients with suspected CMD were prospectively enrolled between September 2024 and March 2025 and examined at the first affiliated hospital of University of Science and Technology (USTC). The selection of CCTA or invasive coronary angiography was determined by cardiologists based on patient symptoms and coronary risk factors. Inclusion criteria for CMD included: (1) symptoms of myocardial ischemia and (2) non-obstructive coronary arteries (defined as <50% stenosis diameter). Exclusion criteria included: (1) presence of myocardial scar on late gadolinium enhancement (LGE), (2) history of coronary revascularization (including percutaneous coronary intervention or coronary artery bypass graft), (3) acute myocardial infarction, (4) atrial fibrillation, (5) severe valvular heart disease, (6) heart failure, (7) cardiomyopathy, (8) chronic kidney disease (eGFR < 45 mL/min), and (9) contraindications to intravenous regadenoson or CMR. All participants were specifically advised to avoid caffeine for 24 h before imaging. Ethics approval was obtained from the Ethics Committee of The First Affiliated Hospital of USTC of China for study procedures, and written informed consent was obtained from all participants prior to participation.

### 2.2 Cardiac PET-MRI protocol

All participants underwent an overnight fast of at least 6 h, except for water intake. PET-MRI scans were acquired on a 3.0 T PET-MRI system (Biograph mMR, Siemens Healthineers, Erlangen, Germany). Each patient underwent simultaneous ^13^N-ammonia PET and gadoterate dimeglumine (Gd-DTPA) perfusion MRI scans at rest and during regadenoson-induced hyperemia.

All patients performed the rest imaging first, followed by stress imaging approximately 1 h later. For rest PET-MRI imaging, a 10 min dynamic PET perfusion scan during rest was started simultaneously with the administration of 370–555 MBq of ^13^N-ammonia (using a second intravenous cannula) followed by a bolus of saline (20 mL at 3 mL/s). MRI perfusion imaging was conducted concurrently with the PET scan. After the start of the PET scan, a single bolus of Gd-DTPA contrast agent (Beilu Pharmaceutical, Beijing, China) (0.075 mmol/kg body weight) was administered via a power injector at a flow rate of 4 mL/s into an antecubital vein, immediately followed by a 20 mL normal saline flush. A saturation recovery turboFLASH CMR sequence was used for MRI perfusion imaging using electrocardiography (ECG)-triggered and breath holding technique. Key parameters included: TR/TE = 2.67/1.15 ms, flip angle of 20°, field of view (FOV) = 380 × 304 mm, slice thickness = 8 mm, matrix size 128 × 102, and a pre-pulse delay of 100 msec.

Stress imaging employed identical parameters to the rest perfusion sequence described above, except that regadenoson was administered 60–90 s prior to simultaneous intravenous injections of ^13^N-ammonia and Gd-DTPA. Regadenoson (400 μg) was administered via an antecubital cannula as a single intravenous bolus over less than 10 s, immediately followed by 5 mL saline flush. Dynamic PET images were reconstructed using a three-dimensional ordered-subset estimation-maximization (3D-OSEM) with 6 iterations and a 5 mm post-reconstruction filter. The dynamic frame protocol included 12 × 10 s, 2 × 30 s, 4 × 60 s and 1 × 180 s intervals using the two-point Dixon attenuation correction.

### 2.3 Image analysis

Left ventricular myocardial perfusion analysis was performed using the standardized 16-segment model defined by the American Heart Association (15). Myocardial blood flow was quantified for both the entire left ventricle and three myocardial regions corresponding to the coronary artery territories (LAD, left anterior descending; LCX, left circumflex artery; and RCA, right coronary artery). CFR was calculated as the stress-to-rest MBF ratio and analyzed at both global and regional levels, respectively.

Image analysis was independently performed by two reviewers. If there was a disagreement between the two, a third observer joined the evaluation. The final result was determined by consensus among all three. Interobserver agreement for image analysis was assessed using kappa coefficient for categorical variables and intraclass correlation coefficient (ICC) for continuous variables. A kappa value >0.75 and ICC > 0.80 were considered to indicate excellent agreement. Regions of interest were defined in the MRI images using CVI42 post-processing software (version 5.1.1, Calgary, Canada). Endocardial and epicardial contours of the left ventricle were manually traced to derive rest and stress data, with subsequent generation of time-signal intensity curves for myocardial tissue and cardiac blood pool. Concordance between CMR and PET was evaluated on a per-vessel basis.

All PET data were quantified semi-automatically using a commercially available dedicated software package (Syngo MBF®, Siemens Healthcare, Erlangen, Germany), generating MBF-values for the entire left ventricle and in three regions corresponding to the coronary artery territories. Mean CFR was calculated as the ratio of stress MBF (stress scan) to rest MBF (rest scan) in each region and globally.

### 2.4 Statistical analysis

Continuous variables were expressed as mean ± standard deviation (SD) or median with interquartile range (IQR). Categorical variables were expressed as frequency with percentage. Pearson’s correlation was used to quantify association between continuous variables. Bland–Altman analysis was performed to evaluate the agreement between CMR and PET. Diagnostic performance of CMR indices for detecting CMD was evaluated through receiver-operating characteristic (ROC) curve analysis. Statistical analyses were conducted using the IBM SPSS Statistics v26.0 (SPSS Inc., Chicago, IL), GraphPad Prism v9.0 (GraphPad Software, La Jolla CA), and MedCalc v19.1 (MedCalc Software, Ostend, Belgium). For all analyses, a two-tailed *p*-value < 0.05 was considered significant.

## 3 Results

### 3.1 Baseline characteristics

**Figure 1:**
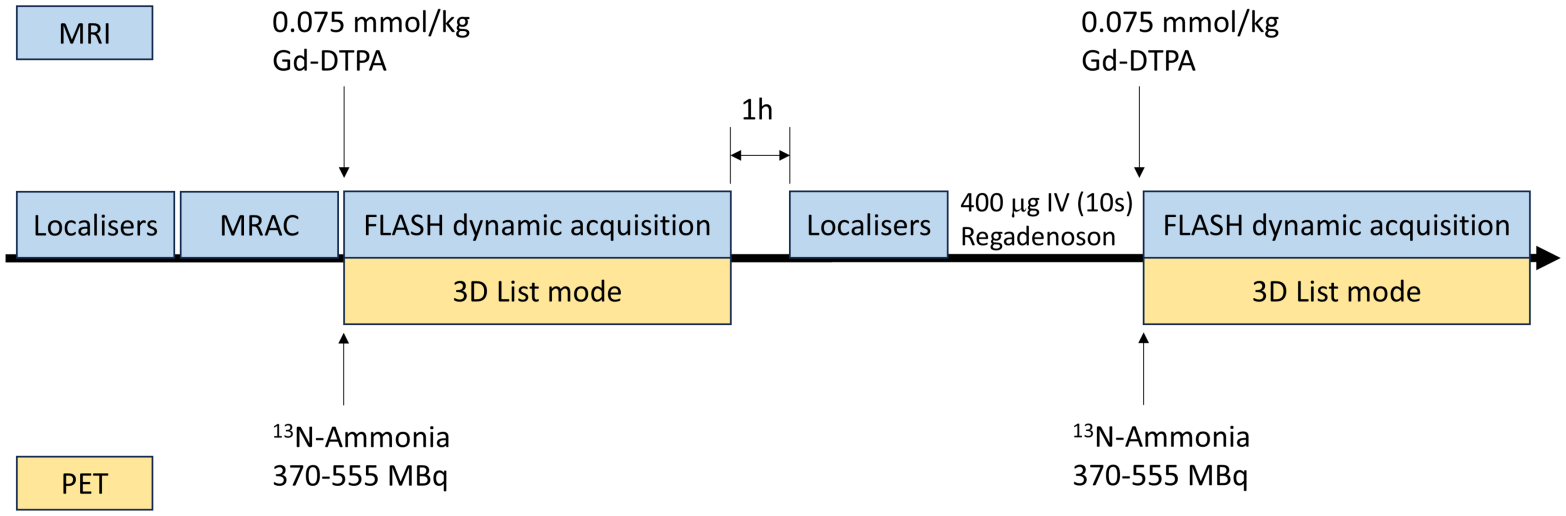
Protocol for simultaneous PET-MRI perfusion imaging. PET, positron emission tomography; MRI, magnetic resonance imaging; MRAC, magnetic resonance attenuation correction; FLASH, fast low-angle shot.

Baseline characteristics and hemodynamic parameters of the cohort of 30 patients (11 were classified as CMD and 19 as the reference group with preserved CFR) are summarized in Tables 1, 2, respectively. The protocol for the study is illustrated in Figure 1.

**Table 1:** Patient characteristics.

Characteristic	Total (*n* = 30)	Preserved CFR (*n* = 19)	Impaired CFR (*n* = 11)	*p*-value
Age (years)	54.4 ± 8.8	53.9 ± 9.5	55.3 ± 7.7	0.686
Female	13 (43.4)	8 (42.1)	5 (45.5)	1.000
BMI (kg/m^2^)	24.3 ± 2.6	23.6 ± 2.4	25.4 ± 2.7	0.074
Stress HR (bpm)	99.6 ± 13.1	101.3 ± 14.6	96.7 ± 9.7	0.369
Rest HR (bpm)	74.2 ± 9.6	74.2 ± 9.6	74.4 ± 9.9	0.956
SBP (mm Hg)	133.1 ± 12.8	130.8 ± 10.9	137.0 ± 15.2	0.208
DBP (mm Hg)	82.1 ± 9.3	81.9 ± 10.1	82.5 ± 8.3	0.857
Symptomatic status				0.637
Typical angina	13 (43.3)	7 (36.78)	6 (54.5)	
Atypical angina	9 (30)	6 (31.6)	3 (27.3)	
Asymptomatic	8 (26.7)	6 (31.6)	2 (18.2)	
Medication				
ACEI or ARB	5 (16.7)	3 (15.8)	2 (18.2)	1.000
Aspirin	13 (43.4)	8 (42.1)	5 (45.5)	1.000
Beta-blockers	10 (33.3)	5 (26.3)	5 (45.5)	0.425
CCB	10 (33.3)	5 (26.3)	5 (45.5)	0.425
Nitrates	7 (23.3)	2 (10.5)	5 (45.5)	0.068
Statins	17 (56.7)	10 (52.6)	7 (63.6)	0.708
Oral hypoglycemic agents	3 (10.0)	0 (0.0)	3 (27.2)	0.041
Laboratory values				
Total Cholesterol, mmol/L	4.55 ± 1.19	4.51 ± 0.86	4.60 ± 1.66	0.868
Triglyceride, mmol/L	1.18 ± 0.58	0.91 ± 0.40	1.66 ± 0.54	<0.001
HDL cholesterol, mmol/L	1.28 ± 0.43	1.33 ± 0.41	1.20 ± 0.47	0.425
VLDL cholesterol, mmol/L	0.61 ± 0.24	0.55 ± 0.19	0.71 ± 0.30	0.081
LDL cholesterol, mmol/L	2.58 ± 0.90	2.60 ± 0.80	2.56 ± 1.09	0.918
WBC, 10^9^/L	6.03 ± 1.84	5.82 ± 1.80	6.40 ± 1.95	0.417
PLT, 10^9^/L	225.6 ± 54.4	224.4 ± 54.2	227.7 ± 57.4	0.874
Neutrophil, 10^9^/L	3.77 ± 1.20	3.83 ± 1.30	3.65 ± 1.06	0.704
Monocyte, 10^9^/L	0.42 ± 0.16	0.42 ± 0.15	0.43 ± 0.18	0.957
Lymphocyte, 10^9^/L	1.98 ± 0.78	1.88 ± 0.52	2.17 ± 1.10	0.418
Hemoglobin, g/dl	137.3 ± 13.9	137.5 ± 14.89	136.9 ± 12.68	0.909
RBC, 10^12^/L	4.61 ± 0.53	4.65 ± 0.42	4.55 ± 0.69	0.626

* Values are mean ± SD or *n* (%).ACEI, angiotensin-converting-enzyme inhibitors; ARB, angiotensin receptor blocker; BMI, body mass index; CCB, calcium-channel blocker; HDL, high-density lipoprotein; VLDL, very-low-density lipoprotein; LDL, low-density lipoprotein; DBP, diastolic blood pressure; SBP, systolic blood pressure.

**Table 2:** Patient hemodynamic parameters.

Variables	MRI	^13^N-ammonia PET	*p*-value
Left ventricular end-systolic volume (mL)	46.90 ± 10.78	36.72 ± 12.58	<0.001
Left ventricular end-diastolic volume (mL)	117.28 ± 21.25	90.34 ± 30.63	<0.001
Left ventricular ejection fraction (%)	60.76 ± 7.34	62.01 ± 8.74	0.501
Global perfusion			
CFR	1.77 ± 0.49	2.56 ± 1.08	<0.001
Regional perfusion			
CFR	1.81 ± 0.62	2.55 ± 1.09	<0.001

* Values are mean ± SD. LV, left ventricle; PET, positron emission tomography; MRI, magnetic resonance imaging; CFR, coronary flow reserve.

Although sex, BMI, and age did not differ significantly between the two study groups, patients with impaired CFR tended to be older and had higher BMI values compared to those with preserved CFR. The study group had a higher incidence of diabetes, hypertension, and hyperlipidemia than the control subjects.

### 3.2 Global myocardial perfusion

**Figure 2:**
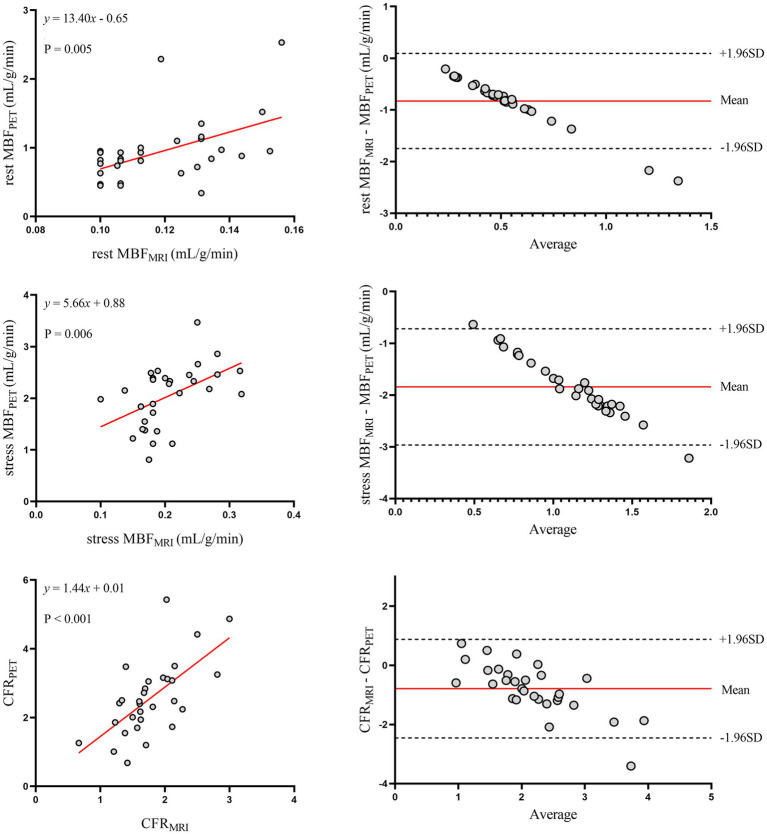
Global myocardial perfusion. Left panels display scatter plots and right panels show Bland–Altman plots comparing MRI and ^13^N-ammonia PET measurements of MBF at rest (top), under stress (middle), and CFR (bottom) on a global basis. In the Bland–Altman plots, the solid red line indicates the mean bias, and the dashed black lines indicate +1.96SD and −1.96SD, respectively. PET, positron emission tomography; MRI, magnetic resonance imaging; MBF, myocardial blood flow; CFR, coronary flow reserve.

Figure 2 shows the relationship between MRI and PET measurements of global myocardial perfusion. On a per patient basis, MRI-derived CFR (CFR_MRI_) and PET-derived CFR (CFR_PET_) showed moderate correlation (*r* = 0.64; *p* < 0.001) and moderate inter-method reliability (ICC for absolute agreement = 0.66 [95% confidence interval (CI): 0.28 to 0.84]; *p* = 0.003). Bland–Altman analysis demonstrated a mean bias of − 0.79 ± 0.85 for CFR. Table 2 (the second to last row) displays the mean values of global CFR as measured using MRI and PET. Global CFR was significantly lower for MRI in comparison to PET (1.77 ± 0.49 vs. 2.56 ± 1.08; *p* < 0.001). MRI-derived MBF (MBF_MRI_) and PET-derived MBF (MBF_PET_) obtained during rest and stress showed correlation (*r* = 0.50, *p* = 0.005 and *r* = 0.49, *p* = 0.006, respectively). Bland–Altman analysis demonstrated a mean bias of −0.83 ± 0.47 mL/g/min and −1.84 ± 0.57 mL/g/min for rest and stress MBF, respectively.

### 3.3 Regional myocardial perfusion

**Figure 3:**
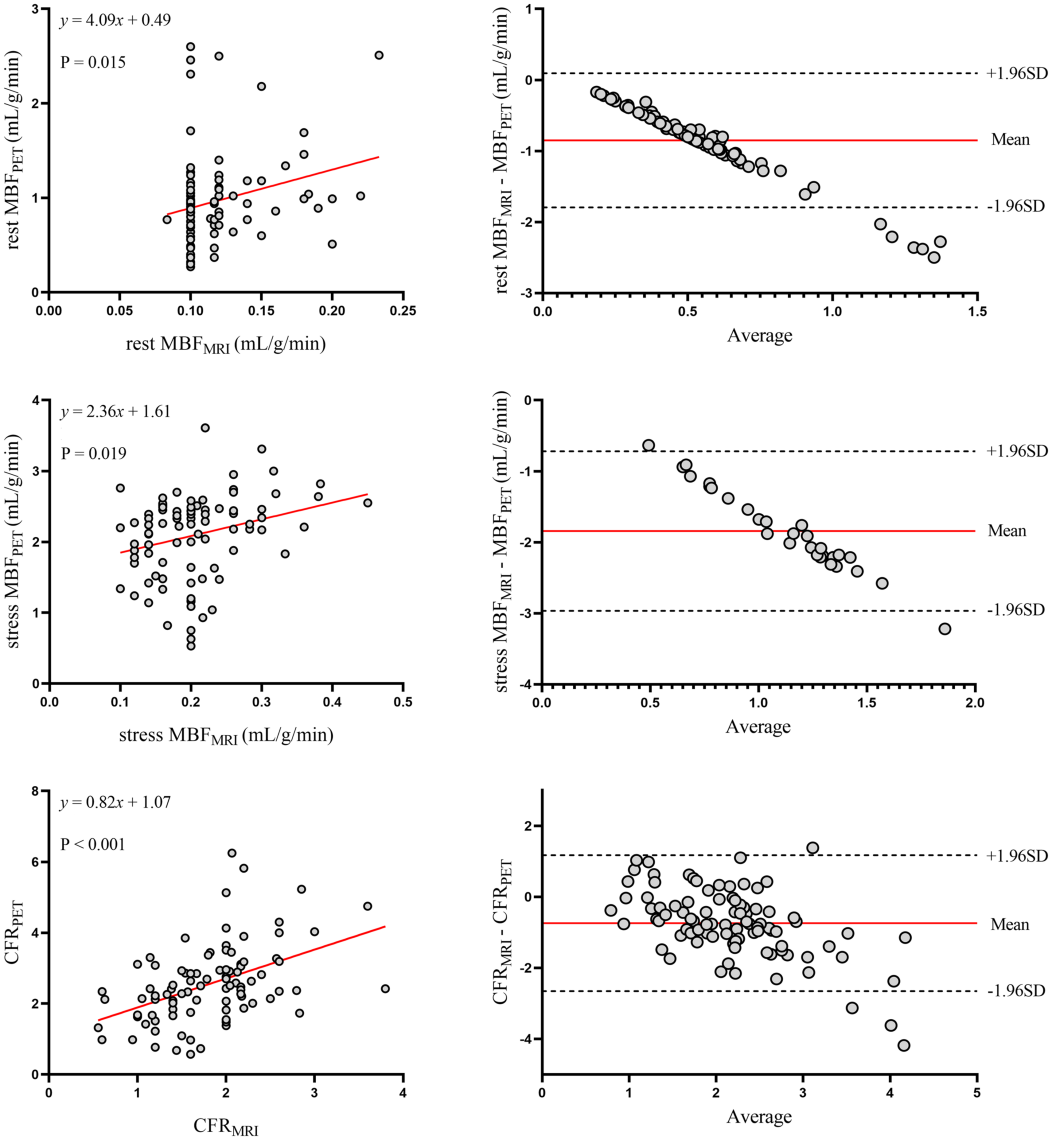
Regional myocardial perfusion. Left panels display scatter plots and right panels show Bland–Altman plots comparing MRI and ^13^N-ammonia PET measurements of MBF at rest (top), under stress (middle), and CFR (bottom) on a per-vessel basis. In the Bland–Altman plots, the solid red line indicates the mean bias, and the dashed black lines indicate +1.96SD and −1.96SD, respectively. PET, positron emission tomography; MRI, magnetic resonance imaging; MBF, myocardial blood flow; CFR, coronary flow reserve.

The relationship between MRI and PET measurements of regional myocardial perfusion is illustrated in Figure 3. On a per vessel basis, correlation (r = 0.48; *p* < 0.001) and inter-method reliability (ICC for absolute agreement = 0.57 [95% CI: 0.34 to 0.71]; *p* < 0.001) were present between CFR_MRI_ and CFR_PET_. Bland–Altman analysis revealed a mean bias of − 0.74 ± 0.98 for CFR. MRI demonstrated a tendency to underestimate CFR at both patient and vessel levels. Table 2 (the bottom row) lists the mean values of MRI and PET measurements of CFR. CFR_MRI_ was significantly lower than CFR_PET_ (1.81 ± 0.62 vs. 2.55 ± 1.09; *p* < 0.001). MBF_MRI_ and MBF_PET_ obtained during rest and stress showed only poor to moderate correlation (*r* = 0.26, *p* = 0.015 and *r* = 0.43, *p* = 0.019, respectively). Bland–Altman analysis showed a mean bias of − 0.85 ± 0.48 mL/g/min and − 1.89 ± 0.64 mL/g/min for rest and stress MBF, respectively.

**Figure 4:**
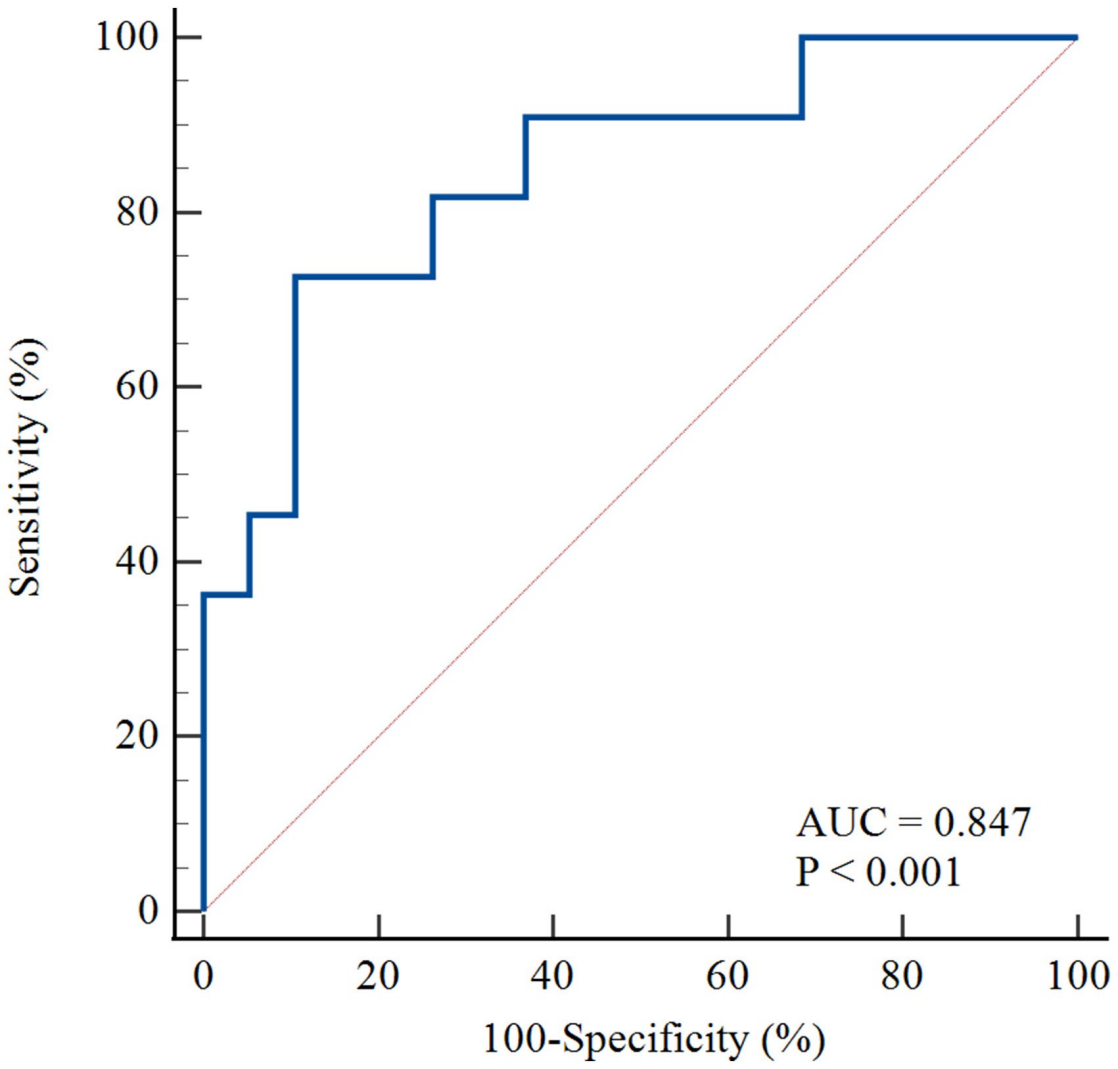
The ROC curve for the detection of abnormal perfusion. The ROC curve of MRI derived CFR for detecting abnormal perfusion defined as 13 N-ammonia PET-derived CFR < 2.0. AUC, area under the curve; ROC, receiver operating characteristic; PET, positron emission tomography; MRI, magnetic resonance imaging; MBF, myocardial blood flow; CFR, coronary flow reserve.

**Figure 5:**
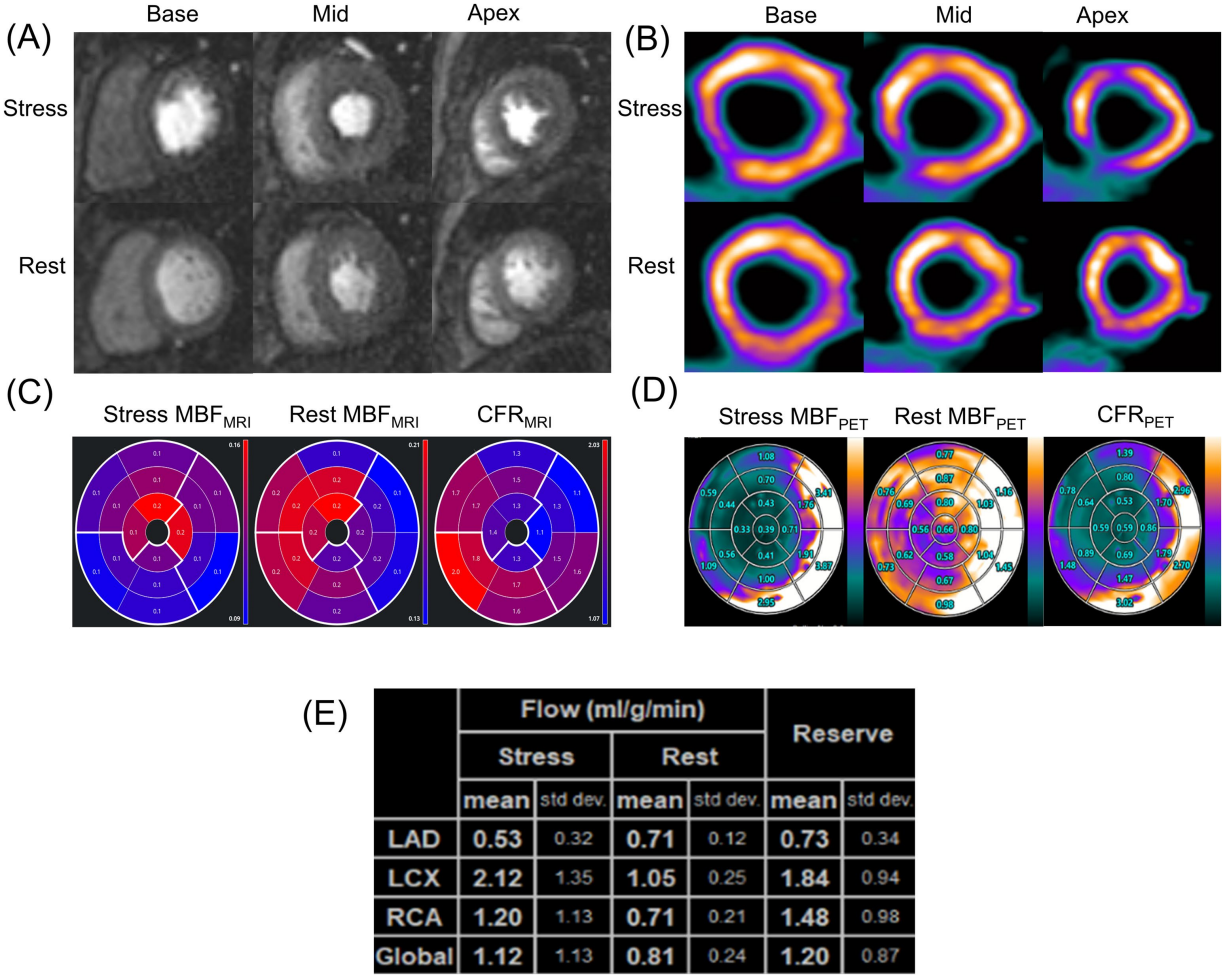
A representative case of a symptomatic 39-year-old man in the impaired CFR group. **(A)** First-pass perfusion images at rest and stress. **(B)** ^13^N-ammonia PET images at rest and stress. **(C)** MRI-derived quantification value of stress MBF (left), rest MBF (center) and, CFR (right). **(D)** Regadenoson-induced ^13^N-ammonia PET-derived quantification value of stress MBF (left), rest MBF (center) and, CFR (right) in the 17-segment standard American Heart Association model and **(E)** based on the three major coronary territories. PET, positron emission tomography; MRI, magnetic resonance imaging; MBF, myocardial blood flow; CFR, coronary flow reserve.

Figure 4 shows the ROC curves of MRI perfusion imaging for detecting impaired CFR as defined by ^13^N-ammonia PET. CFR_MRI_ displayed an area under the curve (AUC) of 0.847 (95% CI: 0.669 to 0.952) and an optimal cutoff value of 1.57 (sensitivity: 72.73%; specificity: 89.47%). Figure 5 shows a 39-year-old symptomatic male in the impaired CFR group. First-pass MRI (Panel A) reveals uniform rest perfusion but diffuse mid-apical signal reduction under stress, consistent with microvascular dysfunction. Corresponding ^13^N-ammonia PET (Panel B) shows matching stress tracer uptake defects. Quantitative analysis (Panels C–E) demonstrates moderate agreement in global CFR (MRI: 1.20; PET: 1.48), aligning with the cohort’s overall correlation (*r* = 0.64), while stress MBF differs more notably (MRI: 1.12 vs. PET: 2.12 mL/g/min), reflecting the broader trend of larger MBF discrepancies. This case exemplifies the study’s key findings: consistent CFR for CMD diagnosis despite variable absolute MBF.

### 3.4 Comorbidity subgroup analyses

**Figure 6:**
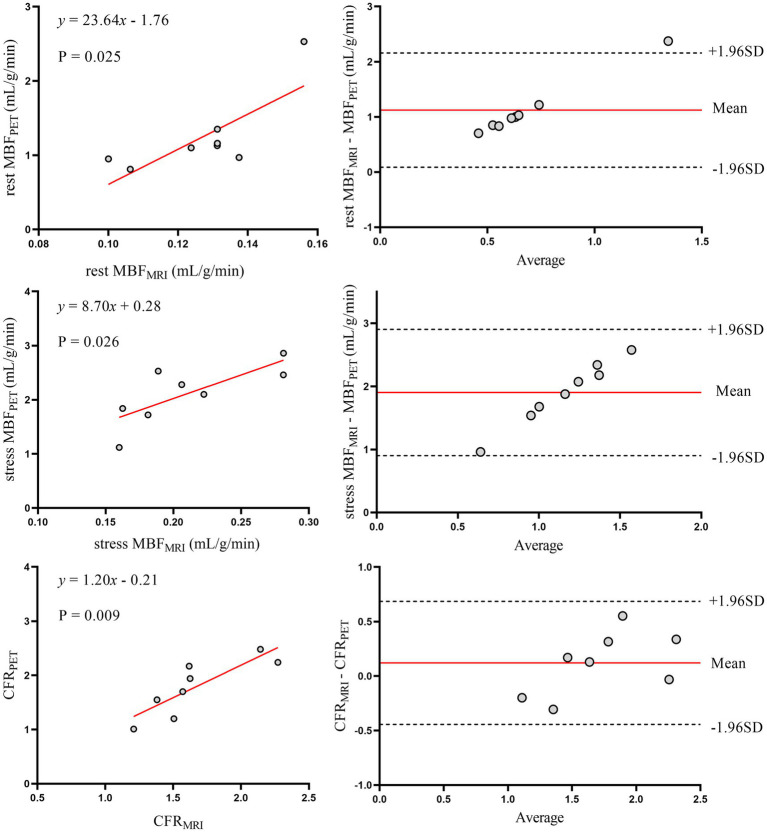
Correlation and agreement of myocardial perfusion metrics (CFR, rest/stress MBF) between MRI and ^13^N-ammonia PET in patients with diabetes mellitus. Left panels (top to bottom): scatter plots with linear regression for rest MBF, stress MBF, and CFR between MRI and PET in patients with diabetes. Right panels: Bland–Altman plots illustrating agreement, with solid red lines denoting mean bias and dashed lines representing 95% limits of agreement (LoA). PET, positron emission tomography; MRI, magnetic resonance imaging; MBF, myocardial blood flow; CFR, coronary flow reserve.

**Figure 7:**
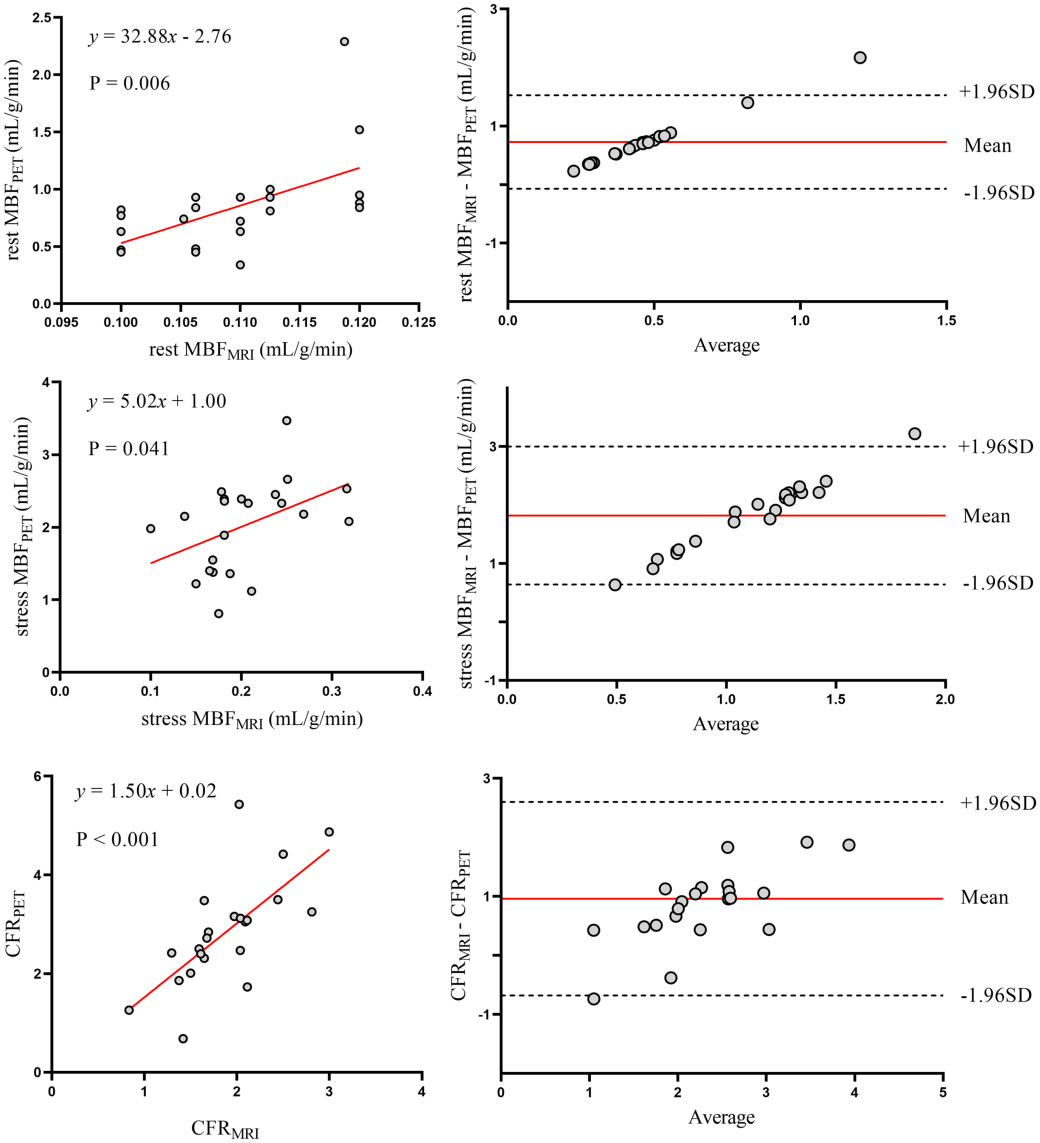
Correlation and agreement of myocardial perfusion metrics (CFR, rest/stress MBF) between MRI and ^13^N-ammonia PET in patients without diabetes mellitus. Left panels (top to bottom): scatter plots with linear regression for rest MBF, stress MBF, and CFR between MRI and PET in patients without diabetes. Right panels: Bland–Altman plots for agreement. PET, positron emission tomography; MRI, magnetic resonance imaging; MBF, myocardial blood flow; CFR, coronary flow reserve.

To further explore the impact of comorbidities, and given that diabetes was the only condition associated with a significant CFR difference (Table 3), we conducted subgroup analyses in patients with and without diabetes (Figures 6, 7). Despite the known microvascular alterations in diabetes, the overall correlation between MRI and PET for CFR persisted, though myocardial blood flow (MBF) measurements exhibited greater variability in diabetic patients—aligning with characteristics of diabetic microvascular dysfunction. Importantly, the diagnostic performance of MRI-derived CFR (using the established cutoff of 1.57 for CMD) remained robust across both subgroups. These results indicate that while diabetes influences microvascular perfusion (evident in MBF variability), it does not negate the core agreement between MRI and PET for evaluating global CFR, thus supporting the primary findings.

**Table 3:** Comparison of CFR between patients with and without hypertension, dyslipidemia, or diabetes.

Comorbidity	Hypertension		Dyslipidemia		Diabetes	
	With	Without	With	Without	With	Without
CFR	2.24 ± 0.76	2.81 ± 1.24	2.44 ± 1.43	2.61 ± 0.93	1.79 ± 0.52	2.84 ± 1.10
*p*-value	0.154		0.692		0.015	

* Values are mean ± SD.CFR, coronary flow reserve.

## 4 Discussion

The current study represents the largest investigation to date of the agreement between MRI and PET measurements of myocardial perfusion. In this study, the feasibility of simultaneous CFR quantification using hybrid PET-MRI scanner was confirmed in a cohort of patients with suspected CMD, setting the stage for a future clinical study to compare PET and MRI CFR values in suspected CMD. While consistency was only moderate with substantial variability, strong correlations and negligible bias were observed between the two methods for both global and regional myocardial perfusion. Thus, in clinical settings, MRI for quantifying myocardial perfusion is an attractive alternative to PET for CMD diagnosis.

PET is widely regarded as the non-invasive gold standard for MBF quantification (16). Despite its short half-life (9.96 min), ^13^N-ammonia remains the gold standard perfusion tracer due to its balanced practicality and image quality—attributes amplified by short positron range and myocardial retention—and is ideally suited for validating novel real-time CMR perfusion techniques with automated quantification potential for clinical implementation (17, 18). Such a study could accelerate acceptance of CMR perfusion as a viable alternative for MBF measurements, enabling broader adoption without the need for ionizing radiation and costly radiochemistry facilities. Additionally, it provides information on left ventricular function and viability, making CMR well-suited for non-invasive CMD evaluation.

CFR is a commonly used measure in the diagnosis of CMD. In our results, CFR values were generated within the normal ranges reported in the literature, both for MRI (19) and for PET (20, 21). Previous studies comparing quantitative CMR and PET myocardial perfusion were limited to patient cohorts, with substantial variability in study populations (i.e., typical angina patients vs. healthy volunteers), PET radiotracers used, CMR image acquisition techniques, and CMR field strength. Engblom et al. (14) showed a strong correlation for CFR values (*r* = 0.92, *p* < 0.001) quantified by PET and MRI. Qayyum et al. (22) reported that, on a global and vessel territorial basis, MRI results were strongly correlated with PET results for CFR (*r* = 0.89, *p* < 0.001). Pack et al. (23) studied 4 healthy volunteers with ^13^N-ammonia PET and CMR perfusion imaging at 3-T and reported similar results. Morton et al. (12) compared quantitative CMR and PET myocardial perfusion in 41 patients with known or suspected CAD, finding strong correlations between CMR-and PET-derived MPRI (*r* = 0.75, *p* < 0.001) despite weak correlations in absolute perfusion values. These findings are consistent with our observations. In our data, there was good correlation between MRI-and PET-based CFR values (*r* = 0.64, *p* < 0.001). However, Kero et al. (24) shown that there was no significant correlation between MRI-and PET-derived CFR values (*r* = 0.08, *p* = 0.80), which in this case can be explained by physiological differences between MRI and PET.

Accurately quantifying MBF with MRI remains challenging due to the non-linear relationship between signal intensity and gadolinium contrast concentration. Key sources of non-linearity and bias include spatial signal variations due to surface coil sensitivity profiles, incomplete saturation of magnetization during contrast agent bolus passage, T2* decay from high contrast concentrations in the blood pool, and inherent non-linear signal responses resulting from saturation recovery dependent on imaging protocol parameters (25). In our study, a 0.075 mmol/kg dose of Gd-DTPA was administered, with no evidence of bolus peak flattening due to saturation effects observed. To avoid signal saturation effects, low contrast agent doses (0.05 mmol/kg) have been used in some studies (26, 27). Similarly, no evidence of bolus peak flattening due to saturation effects was observed. If saturation effects are present but overlooked, myocardial perfusion results may be overestimated, which was not evident in our findings.

MRI and PET showed significant differences in left ventricular functional parameters, with MRI measuring larger end-diastolic volumes (117.28 ± 21.25 mL vs. 90.34 ± 30.63 mL, *p* < 0.001) and end-systolic volumes than PET, though ejection fractions were comparable (Table 2). Clinically, these discrepancies matter because left ventricular volumes are key for assessing cardiac remodeling and disease progression. MRI, with superior soft tissue resolution and breath-hold acquisition, likely provides more accurate volumetric data, while PET may underestimate volumes due to motion artifacts from free breathing or attenuation correction differences. For CMD patients, where subtle ventricular remodeling could relate to microvascular dysfunction, these differences highlight the need to interpret volume-based metrics cautiously when switching between modalities—MRI may be preferred for tracking structural changes, while PET remains valuable for perfusion-focused assessments.

Regarding characteristics of our study population, we have demonstrated that in 30 patients with suspected CMD undergoing myocardial perfusion PET, 36.7% had lower global CFR (< 2.0). The unique relationship between symptoms and impaired CFR highlighted no statistical difference in symptomatic status between the two groups, with approximately 18.2% of the impaired CFR group being asymptomatic. Patients without obstructive coronary artery disease are often told their chest pain is noncardiac. As a result, further evaluation is frequently deferred, leading to missed opportunities to diagnose CMD and initiate treatments to reduce cardiovascular risk. Similar to previous findings (28), a more rapid MFR decline has been observed in diabetic patients compared to non-diabetic counterparts in the presence of significant obstructive epicardial disease. We further analyzed comorbidities. Only diabetes was linked to significant CFR differences (Table 3). Even so, diabetes did not break the core agreement between MRI and PET for global CFR. MBF measurements showed slightly more variability in diabetic patients. But the diagnostic value of MRI-derived CFR for CMD stayed strong in both diabetic and non-diabetic subgroups (Figures 6, 7). Given that diabetes-related differences did not nullify the primary findings on CFR agreement or diagnostic utility, additional model adjustments to correct for these confounding factors were not deemed necessary. Overall, implications for the diagnosis and management of CAD patients with these and other traditional cardiovascular risk factors for microvascular disease have been highlighted by this observation.

In this current study, MRI-derived CFR values underestimated PET values in CMD patients, particularly at higher values, and these findings are in keeping with previous clinical studies that compared PET and CMR-derived CFR (11). There are several plausible reasons to explain the underestimation of CMR-derived MBF values observed in the phantom studies and the volunteer study. MRI signal intensity and gadolinium concentration demonstrate a non-linear relationship (26). This is primarily attributed to the kinetic properties of gadolinium-based contrast agents. The extraction fraction of gadolinium-based contrast agents decreases unpredictably from approximately 0.55 at rest with increasing flow rates, leading to underestimation of tissue response curves at higher flows and subsequent underestimation of CFR. PET MBF quantification avoids this limitation as the activity measured directly correlates with tracer concentration.

Figure 5’s representative case validates the study’s methodology and findings. It demonstrates simultaneous PET-MRI feasibility, eliminating inter-scan variability to strengthen correlation reliability. The case visually confirms core results: CFR alignment and stress MBF discrepancies (mirroring cohort trends, likely due to contrast kinetics). Clinically, it links imaging findings to symptomatic CMD—diffuse stress defects without obstructive disease—bridging quantitative data and real-world diagnosis, reinforcing MRI’s value for identifying microvascular dysfunction.

### 4.1 Study limitations

First, the study enrolled relatively small sample size. Nevertheless, this is the first study in patients and larger than previous volunteer-based studies. Second, respiratory control differences—MRI requiring breath-holds versus PET allowing free breathing—may introduce variability in MBF quantification. MRI breath-holds minimize motion artifacts, improving spatial resolution for regional MBF analysis, but brief breath-holding could transiently alter hemodynamics (e.g., subtle heart rate changes) that affect stress-induced hyperemia. In contrast, PET’s free-breathing approach avoids such transient physiological shifts but may introduce motion-related blurring, particularly in inferior myocardial segments, potentially diluting regional MBF accuracy. These differences likely contribute to the observed discrepancies in MBF between modalities, as motion artifacts and physiological perturbations disproportionately affect absolute perfusion values. Third, while PET MBF and CFR analysis are performed robustly and automatically within minutes using commercial software, MRI analysis remains time-consuming, operator-dependent, and error-prone. Finally, CFR interpretation should be exercised with caution when assigning myocardial segments to coronary artery territories based on common vascular distribution patterns. However, interindividual variability in coronary anatomy may render such assumptions potentially inaccurate.

## 5 Conclusion

The CFR_PET_ and CFR_MRI_ seem to predict CMD equally well and accurately. However, in patients, the absolute perfusion values from PET and MRI are only modestly correlated, indicating the need for further refinement of quantitative techniques.

## Data Availability

The original contributions presented in the study are included in the article/supplementary material, further inquiries can be directed to the corresponding authors.
